# A Case Report of Spontaneous Closure of a Posttraumatic Arterioportal Fistula

**DOI:** 10.1155/2013/623704

**Published:** 2013-12-18

**Authors:** Hirotada Kittaka, Hiroshi Akimoto, Keitaro Tashiro

**Affiliations:** Department of Emergency, Osaka Mishima Emergency Critical Care Center, 11-1 Minami Akutagawa-cho, Takatsuki, Osaka 569-1124, Japan

## Abstract

As the indications for the nonoperative management (NOM) of hepatic injury have expanded, the incidence of complications of NOM has increased. Among such complications, arterioportal fistula (APF) formation is rare, although dangerous, due to the potential for portal hypertension. Embolization is performed in APF patients with clinical signs suggestive of portal hypertension. Meanwhile, no indications for treatment have been established in APF patients without symptoms, as the natural history of posttraumatic APF is not well understood. We herein report the case of a 35-year-old female with severe hepatic injury (Grade IV on the Organ Injury Scale of the American Association for the Surgery of Trauma) due to a traffic accident. Her hemodynamic state remained stable, and an enhanced CT scan obtained on admission showed no extravasation of contrast medium, pseudoaneurysm formation, or APF; therefore, NOM was selected. Although the patient's physical condition was stable, an enhanced CT scan obtained 13 days after the injury showed APF in segment 8 of the liver. Although embolization was considered, the APF was not accompanied by portal dilatation suggestive of portal hypertension; hence, strict observation was selected. Consequently, follow-up CT performed on day 58 after the injury revealed spontaneous closure of the APF.

## 1. Introduction

The most common cause of arterioportal fistula (APF) has been reported to be hepatic trauma (28%), followed by iatrogenic procedures (16%), congenital vascular malformation (15%), malignancy (15%), and rupture of splanchnic artery aneurysms (14%) [1]. As the indications for the nonoperative management (NOM) of hepatic trauma injury have expanded, with high reported success rates ranging from 83% to 100% [2–4], the incidence of complications, including APF, posttraumatic pseudoaneurysms, bile leakage, and hepatic abscesses, has increased [3, 5, 6]. APF is rare; however, it is considered to be clinically dangerous due to the possibility of portal hypertension and ultimate rupture of esophageal varices. Therefore, transarterial embolization is usually performed in APF patients with clinical signs, such as splenomegaly or ascites, that are suggestive of portal hypertension [7–9]. On the other hand, no indications for treatment have been established in APF patients without symptoms, as the natural history of posttraumatic APF is not well understood. We encountered a rare case of spontaneous closure of posttraumatic APF detected on follow-up enhanced computed tomography (CT) for blunt liver trauma.

## 2. **Case Report**

**Figure 1:**
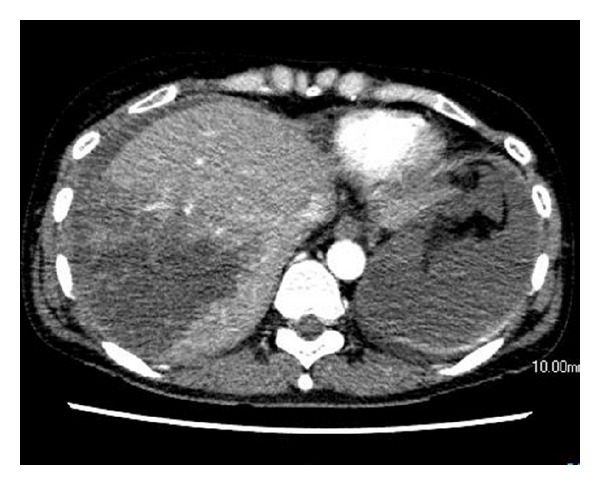
Enhanced CT on admission. Enhanced CT shows a deep liver laceration (Grade IV on the Organ Injury Scale of the American Association for Surgery of Trauma) without extravasation or pseudoaneurysm or arterioportal fistula formation.

**Figure 2:**
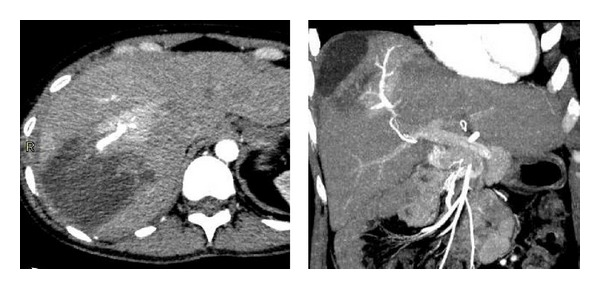
Follow-up CT performed 13 days after the injury. Enhanced CT reveals an arterioportal fistula in segment 8 of the liver with partial enhancement of the liver parenchyma in the early phase.

**Figure 3:**
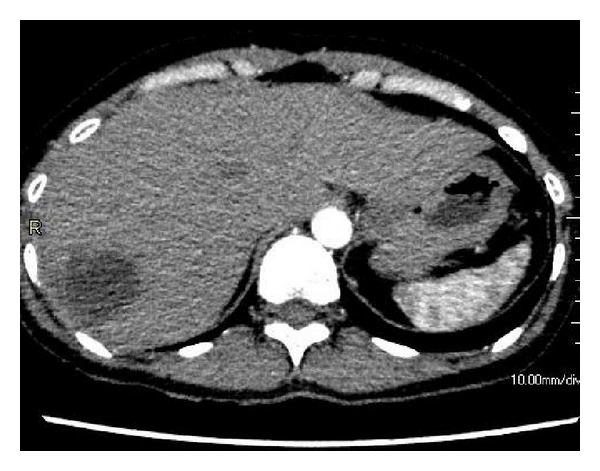
Follow-up CT performed on day 58. The size of the low-density area of the anterior segment of the liver is reduced, and the arterioportal fistula has disappeared.

A 35-year-old female injured in a traffic accident in which a car driving at a speed of 40 miles per hour crashed into a wall was transported to a regional base hospital. Although the patient was hemodynamically stable, an enhanced CT scan revealed a severe liver laceration (Organ Injury Scale of the American Association for the Surgery of Trauma, Grade IV) on the right lobe with intra-abdominal hemorrhage; therefore, she was transferred to our institution eight hours after the injury. Her hemodynamic state remained stable, and an enhanced CT scan performed at our institution showed no extravasation of contrast medium, pseudoaneurysm formation, or APF (Figure 1); hence, NOM without angiography was selected. After admission, the patient's hemodynamic state continued to be stable, and the volume of intra-abdominal hemorrhage evaluated on ultrasonography did not increase. Food consumption was initiated on day 2 of hospitalization, and a follow-up CT scan performed on day 4 revealed no pseudoaneurysms or APF; therefore, the restriction of activities was canceled. No changes were observed in the patient's general condition, and the levels of transaminases, which were highly elevated on admission (AST: 1,810 U/L, ALT: 662 U/L), gradually decreased to within the normal limits. The patient was discharged on day 11 and received regular outpatient treatment. An enhanced CT scan obtained 13 days after the injury showed an intrahepatic APF in segment 8, without pseudoaneurysm formation (Figure 2). Although embolization was considered, the APF was not accompanied by portal dilatation suggestive of portal hypertension; therefore, severe observation was selected. Consequently, spontaneous closure of APF was obtained on follow-up CT performed on day 58 after the injury (Figure 3). Three months later, reexamination with enhanced CT showed no APF or signs of portal hypertension, and all laboratory data were within the normal limits. The patient is currently alive, with no symptoms, six months after the injury.

## 3. Discussion

Over the last three decades, nonoperative management (NOM) of blunt hepatic trauma injuries has become the standard of treatment for hemodynamically stable patients, with a reported success rate of over 80% [4, 5]. According to the Eastern Association for the Surgery of Trauma practice management guidelines, high-grade hepatic injury (Grade IV-V on the Organ Injury Scale of the American Association of Surgery for Trauma) on CT is no longer an absolute contraindication for NOM [10]. While the indications for NOM have expanded to include more severe hepatic injuries, a higher incidence of complications of NOM, such as bile leakage, bile peritonitis, missed injuries, hepatic abscesses, and delayed hemorrhage due to pseudoaneurysm formation, has been reported [3, 5, 6]. APF is a comparatively rare complication of liver injury; however, it can lead to portal hypertension, consequently resulting in gastrointestinal bleeding, mesenteric ischemia, and heart failure [7, 11]. The period between injury and the diagnosis of APF varies considerably, ranging from several days to more than 20 years [8, 12–14]. Most patients diagnosed with APF long after injury exhibit clinical signs of portal hypertension, such as gastrointestinal bleeding, ascites, and splenomegaly. On the other hand, those diagnosed within several days tend to display no symptoms, and most cases are detected accidentally on follow-up imaging examinations. Tanaka et al. [14] also reported that APF was detected in five of 65 hepatic injury cases on follow-up CT scans and that three patients demonstrated spontaneous closure within a few months after the injury. The authors concluded that when APF is small and located peripherally without signs of portal hypertension, spontaneous closure can be expected. Guzman et al. [15] introduced a novel classification of APF in which the disease is classified into Types 1, 2, and 3 depending on the etiology (acquired or congenital), size (large or small), and location (extrahepatic or peripheral or central to the liver). In this classification, most patients with APF categorized as having Type 1 disease, which is usually diagnosed on a percutaneous liver biopsy, are asymptomatic, and the fistulae generally resolve spontaneously within one month. On the other hand, those with Type 2 APF, which is located in the central portion of the liver, should be treated with embolization or a surgical approach due to the potential for portal hypertension and hepatoportal sclerosis. According to this classification, the APF observed in the present case can be categorized as Type 2 considering its etiology and location in the liver; therefore, immediate intervention is advisable. However, conservative therapy without intervention was selected because the APF was comparatively small and was not accompanied by portal dilatation suggestive of portal hypertension. Consequently, spontaneous closure of the APF was obtained two months after the injury.

Further investigations with a large number of patients are required to obtain a deeper understanding of the clinical course of posttraumatic APF without clinical signs of portal hypertension detected accidentally on follow-up imaging examinations and determine the treatment indications for such cases of APF.
